# Gender and Exercise in Relation to Obesity in Greek Elderly Population

**DOI:** 10.3390/ijerph17186575

**Published:** 2020-09-09

**Authors:** Sousana K. Papadopoulou, Dimitrios Papandreou, Elias Tassoulas, Fani Biskanaki, Stavros Kalogiannis, Maria N. Hassapidou

**Affiliations:** 1 Department of Nutritional Sciences & Dietetics, International Hellenic University, 57400 Thessaloniki, Greece; sousana@nutr.teithe.gr (S.K.P.); kalogian@ihu.gr (S.K.); mnhas@nutr.teithe.gr (M.N.H.); 2 Department of Health Sciences, CNHS, Abu Dhabi 144534, UAE; 3 Cardiology Department, Watford General Hospital, Watford WD 18 0HB, UK; eltassou@otenet.gr; 4 Ministry of Education, 15180 Athens, Greece; fani.biskan@gmail.com

**Keywords:** elders, exercise, body fat, sarcopenic obesity, central obesity

## Abstract

Purpose: The prevalence of sarcopenic obesity is increasing in older adults (>65 years) and older. Sarcopenic obesity is also related to reduced muscle synthesis, due to low physical activity levels. The purpose of the present study is to investigate possible risk factors, and effects of habitual activity status on different types of obesity in an elderly population. Methods: One hundred and two (n = 102) free living participants, aged >60 years, were randomly selected from Rehabilitation Centers for the Elderly in Thessaloniki and from municipal gymnasiums of Thessaloniki, Greece with a mean age of 68.11 ± 6.40 years. The response rate of the participants was 51%. For the purpose of this study, all the participants selected were healthy and did not receive any medication. Specifically, 46 subjects (19 men and 27 women) were members of Rehabilitation Centers for the Elderly in Thessaloniki, while 56 individuals (31 men and 25 women were members of the municipal gymnasiums of Thessaloniki and exercised 2 to 3 times per week). Anthropometric measurements were taken for all subjects. Body composition was assessed with bioelectrical impedance. Body Mass Index (BMI) was categorized according to the World Health Organization (WHO) (2000) standards. Central obesity was defined as a waist circumference of >102 cm in men and >88 cm in women. All participants completed a specific questionnaire regarding their health status, physical activity and previous weight status. Risk of sarcopenic obesity was diagnosed in the participants with co-existing sarcopenia and obesity resulting in high fat mass concurrent with low lean body mass. Results: Women had more than double risk of developing abdominal obesity (OR:2.133, 95% CI: 0.963–4.725) compared to men. More specifically, 69.6% of the elders who did not exercise regularly had central obesity (men: 52.6% and women: 81.5%), while 38.2% of the exercised elders (men: 36.7% and women: 40%) had central obesity. Sedentary elders demonstrated an increased risk of obesity according to body fat (%BF) (OR: 1.259, 95% CI: 0.576–2.750), double the risk of obesity according to body mass (OR: 2.074, 95% CI: 0.765–5.622), and triple the risk of having central obesity (OR: 3.701, 95% CI: 1.612–8.494) compared to those who exercised. Conclusion Exercise appears to have a protective role against all modes of obesity and thus possibly against obesity-related co-morbidities in the elderly.

## 1. Introduction

Improvement of socioeconomic circumstances, including nutrition, during the last two centuries has led to health improvements and increased life expectancy in the developed world. The increase in the absolute and relative numbers of the elderly in population is referred to as “population ageing”. However, most significant is the term “successful aging”, meaning not only increased life expectancy but also, and most importantly with a good quality of life; that is, maintenance of health and independence [1]. Ageing is associated with a loss of muscle mass and strength, called sarcopenia, originating from the Greek words sarx (flesh) and penia (loss), meaning “poverty of flesh”. Sarcopenic obesity consists of a disturbance, that combines sarcopenia with obesity. Sarcopenia and obesity in the third age may reinforce each other and act synergistically, maximizing their detrimental effects on disability and mortality [2]. According to Baumgartner [3]. obesity, sarcopenia and sarcopenic obesity can be considered “syndromes of disordered body composition”.

Sarcopenia consists of a progressively increased catabolism and a decreased anabolism with a parallel reduction in the capacity for muscle regeneration [4,5]. There is a loss of motor units that results in a decline in muscle power and predisposes an individual to the development of the metabolic syndrome [4]. The elderly who become frail and obese present with a low relative muscle mass, low muscle strength per muscle area (that is low muscle strength relative to their body size resulting in an increased need to carry a comparatively heavy body), an excess of body fat due to obesity and a parallel increase in weakness due to sarcopenia [6,7] Goodpaster et al. [8] provided evidence that, in the elderly, the decline in muscle strength is higher than the degree of muscle mass loss, thus provoking a deterioration also in muscle “quality”. Inactivity of the elderly, places them at a greater risk for sarcopenia because (a) there is a lack of the normal trophic effect of exercise on muscles and (b) it leads to weight gain, most of which appears to be fat and not free muscle mass [9]. A recent systematic review and meta-analysis conducted in 2019 with combined data of 41 studies and a total of 34,955 participants, concluded that the prevalence of sarcopenia in community-dwelling individuals was 11% of men and 9% of women. Amongst those in nursing-homes individuals sarcopenia was present in 51% of men and 31% of women and the respective gender values of hospitalized individuals was 23% and 24% [10].

Sarcopenia increases from 15% to 40%, between the ages of 60 and 80 years [11]. Similarly, sarcopenic obesity rises with age, and one study found that the prevalence of low lean mass and obesity in the elderly was 33.5% in females and 12.6% in males [12]. However, over the same age range, there was a decrease in obesity prevalence from 55% to 30%. These percentages could be an indication that the eldely maintain their fat mass while losing their muscle mass, and thus obesity may convert to sarcopenic obesity that does not necessarily involve excessive weight gain, among those in their sixties and eighties [13]. A logical explanation was given by Davison et al. [1], suggested a differential survivor effect according to weight status. In other words, the obese elderly had higher mortality rate, compared to non-obese and thus, those above 80 years of age represented an initially healthier group in comparison to the younger elderly [1,12,14].

This population group has the most rapid growth and also is the most vulnerable to the development of several diseases such as diabetes, cardiovascular diseases, stroke, osteoporosis and dementia. Physical activity has a positive effect on the cardiovascular, respiratory, gastrointestinal and musculoskeletal systems [15,16] as well as some types of cancers [17]. Current intense aerobic exercise during leisure time such as fast walking, jogging, racket games, cycling or swimming was found to be protective against CHD and heart attacks in middle and older age subjects [16]. Also, physical activity increases basal metabolism and contributes to regulation of appetite, control of body weight, maintenance of ideal body fat and, in the long-term, leads to a reduction of body weight [15,18]. Similarly, moderate-intensity exercise leads to a reduction in prevalence of diabetes [19,20,21,22], hypertension, hypercholesterolemia and cardiovascular disease [23]. From the time of Hippocrates in Greece, and Susruta in India, physical activity has been recognised to be associated with physical and mental health [16].

Subjects above 65 years of age are recommended to follow the lifestyle that is suggested to younger populations [24]. An earlier study, conducted in Thessaloniki Greece, showed that both older males and females elderly had low physical activity levels, that was attributed to their sedentary lifestyles [25]. A similar result reported by Meijer et al. [26], showing that time spent on low level activities (like lying, sitting and standing) was related to low mean physical activity levels in the elderly.

The purpose of the present study is to estimate different modes of obesity in an older Greek population according and their relation to various anthropometric measurements and physical activity levels.

## 2. Materials and Methods

Out of the 200 hundred approached subjects, the study included 102 elderly subjects, 50 men and 52 women, (60–83 years). The mean age of the total sample was 68.11 ± 6.40 years, and participants were free-living and non-institutionalized from Thessaloniki, Northern Greece. Data was collected between May of 2017 and March of 2018. The response rate of the participants in this study was 51%.As an exclusion criteria, all the participants selected were healthy and were not taking any medication. Out of the total sample, 46 subjects (19 men and 27 women) were members of Rehabilitation Centers for the Elderly in Thessaloniki, while 56 persons (31 men and 25 women) were members of the municipal gymnasiums of Thessaloniki and exercised two to three times per week). The participants completed a specific questionnaire regarding their health status, their physical activity and their previous weight status [27]. More specifically, this questionnaire included specific questions in order to specify the health, physical activity and weight status of all the participants when they were 30 years old. The study was approved by the ethical committee of ATEI (No: 20102) and all participants provided a written informed consent form.

Anthropometric measurements were taken from all participants. Body weight was measured with a digital scale of an accuracy of ±100 g (Seca 707, Seca Corporation, Seca, Columbia) and body height was measured to the nearest 0.5 cm using a stadiometer, of 0.5 cm accuracy (SECA 220) and that was calibrated regularly. Body fat was assessed using two methods: (1) skinfold thickness method, and (2) bioelectrical impendence analysis (BIA). For bioelectrical impendence analysis (BIA), a Maltron 907 bioelectrical impendence analyser (Maltron, Rayleigh, Essex, UK) was used. Participants were abstained from exercising, eating or drinking for four hours, and from taking diuretics the previous week before BIA measurements were completed. Participants were asked to urinate 30 min before the measurement. Subjects lay in a supine position with legs and arms abducted. Electrodes were placed on the right hand (proximal to the third metacarpophalangeal joint) and one on the wrist (between the distal prominence of the radius and ulna) and one on the right foot (proximal to the third metatarsophalangeal joint) and one on the ankle (between the medial and the latearal maleoli).

Skinfold measurements were taken from the right side of the body, in two regions: triceps, and subscapular using a Harpenden skinfold caliper (British Indicators Ltd., London, UK). Measurements were carried out in duplicate and the mean recorded value was used, in order to avoid discrepancies of above 10% between duplicate measurements. Body fat was estimated using the specific age population equations [28].

BMI was categorized according to the WHO [29] standards. Body Mass Index (BMI), Fat Mass Index (FMI = Fat Mass (kg)/Height^2^ (m)) and Fat Free Mass Index (FFMI = Fat Free Mass (kg)/Height^2^ (m)), were calculated for each participant. Obesity, based on body mass, was defined as a BMI greater than 30 kg/m^2^. Central obesity was defined as a waist circumference of >102 cm in men and >88 cm in women. Body fat status was categorized using the criteria of Lohman [30]. Muscle mass <9.12 kg/m^2^ for men and <6.53 kg/m^2^ for women was used to identify the risk of sarcopenia [1]. Obesity was defined as body fat (% body weight) >37.16 for the men and >40.01 for the women [1]. Sarcopenic obesity risk was estimated in the participants with coexisting sarcopenia and obesity resulting in high fat mass (>37.16 for men and >40.01 for women) in combination with low lean body mass (<9.12 kg/m^2^ for men and <6.53 kg/m^2^ for women) [31]. Dietary food intake was recorded and analyzed using the Food Processor nutrition program (version 7.4, 1997, ESHA Research, Salem, OR, USA). Intake of micronutrients was expressed as a percentage of the Recommended Dietary Allowance (RDA) in order to estimate the adequacy of micronutrient intake. Verbal and written instructions on how to record the food intake were given by a dietitian.

## 3. Statistical Analysis

Differences in numeric variables according to gender and according to exercise status were assessed using independent samples t-tests whereas differences in the subjects when they were 30 years old were evaluated with paired t-test. Differences in qualitative variables according to gender and exercise were assessed by conducting chi square tests. The odds ratio were calculated using the cross product, however, the CI intervals were based on using chi-square distribution. All analyses were carried out using the Windows-based SPSS Statistical Package (version 17.0; SPSS, Chicago, IL, USA), and *p* values of ≤0.05 were considered statistically significant.

## 4. Results

Table 1 presents the anthropometric characteristics of the sample.

When aged 30 years old, 14 women and 5 men were overweight and none of the participants was obese. The women demonstrated more than twice the risk of abdominal obesity (OR:2.133, CI: 0.963–4.725) compared to men (Table 2).

Between sedentary and exercised elderly (Table 3), the first demonstrated increased odds for obesity according to body fat (%BW) (OR: 1.259, 95% CI: 0.576–2.750), double chances for obesity according to body mass (OR: 2.074, 95% CI: 0.765–5.622), and triple odds for abdominal obesity (OR: 3.701, 95% CI: 1.612–8.494).

Anthropometric characteristics according to gender and exercise are presented at Table 4 while the percentage of obesity, central obesity and elevated fat levels, according to gender and exercise are presented in Table 5.

Energy intake was 1881 ± 436 Kcal for non-exercised elders and 2064 ± 767 Kcal for the exercised group. The contribution for macronutrients in energy intake was for carbohydrates was 41.74 ± 8.76%, for proteins it was 18.43 ± 5.93% and for fat was 39.68 ± 9.25% for the non-exercised elders. For elders in the exercised group the mean intake for carbohydrates was 42.36 ± 10.46%, proteins were 16.36 ± 4.22% and fat was 39.88 ± 11.49%. There was no significant difference between sexes. Both exercised and non-exercised elders had inadequate intake of several micronutrients such as biotin, vitamin D, folic acid, calcium and magnesium (Data not shown).

## 5. Discussion

Women had more that double the risk of developing abdominal obesity (OR: 2.133, 95% CI: 0.963–4.725) compared to men. The present study showed that the FMI of the men in our study surpassed the 95th percentile of the Swiss men whereas the FFMI was lower than the 5th percentile of Swiss men of a similar age [32]. On the other hand, women demonstrated “normality” belonging in the 75th percentile for the FMI and around the 50th for FMI compared with their peers from Switzerland. The concept of FFMI and FMI, has been previously described in adults and elderly individuals [33], as an indicator of nutritional status and excess energy stores (obesity) and is expected to increase with age, as height naturally declines. According to Schutz et al. [32], the high sensitivity of FMI and FFMI to slight changes of body fat stores and lean tissue mass, compared to the use of BMI or percentage body fat, make them indexes of great interest when assessing static and dynamic nutritional status and energy reserves endpoints.

The elderly of this study had similar BMI and body fat values compared to elders in a former study in Northern Greece [27], and they had lower values compared to American elders [13,34]. BMI values above the 85th percentile were correlated with diseases [34].

An interesting finding of the present study is that exercise had a protective role against prevalence of central obesity (*p* < 0.05). Sedentary elderly demonstrated a non-significant increased odds for obesity according to body fat (%BW) (OR: 1.259, 95% CI: 0.576–2.750), double the chances of obesity according to body mass (OR: 2.074, 95% CI: 0.765–5.622), and almost four times the odds for abdominal obesity (OR: 3.701, 95% CI: 1.612–8.494) compared to exercised ones.

Thus, the 69.6% of the elderly who did not exercise regularly had greater central obesity rates (men: 52.6% and women: 81.5%), while the respective percentage for elders who did exercise was 38.2% (men: 36.7% and women: 40%). Similar results were reported by Slattery et al. [35], who found that physical activity was inversely associated with BMI in women and with skinfold-thickness measures in both men and women (black and white). Physical activity was inversely related to central obesity [35,36]. Other studies found an inverse relationship between physical activity and obesity [37,38]. Regular exercise, especially aerobic, has been found to act protectively against central obesity reducing total, abdominal subcutaneous, and visceral fat, as it favours mobilization of lipids from the abdominal region to the femoral region without causing any changes to BMI [39,40]. So, regular exercise can prevent or ameliorate the metabolic and psychological comorbidities including central obesity, hypertension and hypercholesterolaemia [41,42].

Exercise and a healthy diet are the cornerstones of weight management and health [43] Subjects above 65 years of age are recommended to follow the lifestyle that is suggested to younger populations [24]. An earlier study conducted in Thessaloniki showed that both male and female elderly had low physical activity levels, that was attributed to their sedentary lifestyle [25]. A similar result reported by Meijer et al. [26], demonstrated that time spent on low level activities (like lying, sitting and standing) was related to low mean physical activity levels in the elderly. Elderly individuals with central obesity are at high risk of developing cardiometabolic diseases. Exercise appears to have a more critical role than diet as calorie restriction can cause muscle mass decrease [44]. On the contrary, exercise, and especially progressive resistance training can provoke gain in lean body mass through increases in circulating testosterone, growth hormone and Insulin-like Growth Factor (IGF-I) [45]. Additionally elderly undergoing progressive resistance training experienced increased feeling of well-being and had improved bone metabolism. Various types of exercise, like progressive resistance training, aerobic exercise, High-Intensity Interval Training (HIIT), multi-modal exercise, or even passive exercise seemed to have a positive effect on older adults [46].

There is a large body of evidence that several aspects of diet may be important in the development of sarcopenia [47]. Energy intake decreases by approximately 25% from 40–70 years of age, and even more so if combined with a tendency towards a diet that could be described as monotonous, which may lead to inadequate intake of nutrients. In order to fight sarcopenia, energy intake should be higher than 25–30 kcal/kg of body weight. A high- quality protein intake, equally distributed in doses of 20–40g, is crucial to overcome the anabolic resistance to protein intake and increase postprandial muscle protein synthesis [48]. Three areas have been considered as key in respect to diet in sarcopenia: (1) vitamin D, (2) protein (rich in leucine) and (3) antioxidants. High-quality protein from whole foods, as well as dietary supplements providing isolated proteins, such as whey, casein, egg, meat and soy increase accretion of postprandial protein and induce muscle protein synthesis [46,48,49]. Hence, physical activity and nutritional status are some of the major factors that affect body weight and compostion as the prevalence of sarcopenia in nursing homes and hospitalized patients (38% and 23%), who spend a lot of hours in bed and often do not have a choice of what foods to eat is two or three times higher than the community dwelling individuals (10%) that are more physically active and choose their own foods [10].

A former study in elders of Northern Greece showed similar energy intake, lower intake in fat and protein and higher intake in carbohydrates [50]. It is well documented that chronic diseases and their multiple medications increase interaction between medicine and nutrients causing inadequate nutrient absorption and malnutrition. In accordance, morphological and functional changes in vital organs provoke symptoms similar to malnutrition and that is the reason why the latter is not always predictable in the elderly [51]. Physical inactivity and poor physical condition are aggravating factors for morbidity and mortality [52,53], as is a reduction in lean body mass [54]. The findings of the present study need to be replicated in larger cohort studies with measures of functional status.

In the present study we aimed to define all possible modes of obesity within the Greek elderly population. We acknowledge the limitation of the small sample size and the lack of the assessment of the prevalence of diabetes due to the lack of blood sugar and fasting insulin measurement. Another limitation was the age difference between men and women basically due to the sampling process variation. Moreever, the use of BIA was chosen due to the fact that it was convinient to use, and time efficient. However, different cut-off points and validation studies are needed regarding the diagnosis of sarcopenia using BIA [55]. In adition, confounding factors were not addressed in the statistical analysis which can be also considered as another limitation.

## 6. Conclusions

Elderly individuals who were sedentary demonstrated statistically significant increased odds for abdominal obesity compared to those who exercised two-three times a week. Due to the fact that abdominal obesity is strongly associated with chronic disease, especially in the elderly, exercise, along with a healthy diet may be useful modalities for healthy ageing. The results of this analysis, are of great importance with potential clinical impact to public health and the elderly population. Hence, public health authorities could promote exercise, for community dwelling and institutionalized elderly, while health professionals could use exercise as a means of body weight control.

## Figures and Tables

**Table 1:** Anthropometric characteristics of the sample (mean ± SD).

	Women (n = 52)	Men (n = 50)	Total (n = 102)
Body Weight (kg)	71.9 ± 12.7	83.1 ± 12.7 ***	77.4 ± 12.7
Height (m)	1.62 ± 0.06	1.73 ± 0.06 ***	1.67 ± 0.08
BMI (kg/m^2^)	27.6 ± 4.5	27.7 ± 2.9	27.7 ± 3.8
FMI (kg/m^2^)	11.1 ± 2.9	11.0 ± 3.6	11.1 ± 3.2
FFMI (kg/m^2^)	16.5 ± 3.7	16.7 ± 3.6	16.6 ± 3.7
Waist Circumference (cm)	91.5 ± 11.7	100.7 ± 91.5 ***	96.0 ± 11.0
Waist to hip ratio	0.87 ± 0.10	0.97 ± 0.06 ***	0.92 ± 0.10
Body Fat (%BW)	39.9 ± 8.9	39.7 ± 12.0	39.8 ± 10.5
Body Weight at 30 Years (kg)	61.1 ± 6.9	69.9 ± 6.8	65.5 ± 8.1 ^†^
Height at 30 Years (m)	1.62 ± 0.06	1.73 ± 0.06 ***	1.68 ± 0.08 ^‡^
BMI at 30 Years (kg/m^2^)	23.3 ± 2.6	23.3 ± 1.9	23.3 ± 2.3 ^‡^

*** Statistically significant compared with the women (*p* ≤ 0.001).^†^ Statistically significant compared with when aged 30 years old (*p* ≤ 0.01). ^‡^ Statistically significant compared with when aged 30 years old (*p* ≤ 0.001).

**Table 2:** Modes of obesity between women and men (n (%)).

	Women (n = 52)	Men (n = 50)	Total (n = 102)	OR for Women	CI (95%)
Obesity (WHO)	10 (19.2%)	10 (20%)	20	0.976	0.367–2.596
Obesity (NHANES III)	26 (50.0%)	24 (48%)	50	1.083	0.498–2.356
Central Obesity	32 (61.5%)	21 (42.0%)	53	2.133	0.963–4.725

**Table 3:** Modes of obesity between sedentary and exercised elders.

	Sedentary (n = 46)	Exercised (n = 56)	Total (n = 102)	OR for Sedentary	CI (95%)
Obesity (WHO)	12 (26.1%)	8 (14.3%)	20	2.074	0.765–5.622
Obesity (NHANES III)	24 (52.2%)	26 (46.4%)	50	1.259	0.576–2.750
Central Obesity	32 (69.6%)	21 (37.5%)	53	3.701	1.612–8.494

**Table 4:** Anthropometric characteristics according to gender and exercise.

	Non Exercised Elders		Exercised Elders	
	Men (n = 19)	Women (n = 27)	Men (n = 31)	Women (n = 25)
Body weight (kg)	82.74 ± 12.02	75.33 ± 14.19 * ^a^	83.29 ± 8.91	68.16 ± 9.74 * ^a^
Body height (cm)	1.71 ± 0.07 *	1.62. ± 0.58	1.75 ± 0.06	1.61. ± 0.59
Body Mass Index	28.413 ± 0.38	28.55 ± 5.22	27.32 ± 2.61	26.55 ± 3.24
Waist circumference (cm)	102.921 ± 0.29	96.301 ± 2.06 * ^b^	99.37 ± 5.25	86.31 ± 8.97 * ^b^
Hip circumference (cm)	105.36 ± 6.80	108.831 ± 6.07 * ^c^	102.80 ± 3.90	101.62 ± 8.03 * ^c^
Waist to Hip ratio	0.98 ± 0.07	0.89 ± 0.11	0.97 ± 0.05	0.85 ± 0.09
Body fat (%)	25.12 ± 10.35	27.539 ± 0.91	25.81 ± 11.08	32.21 ± 7.18

* *p* < 0.05. ^a,b,c^ Different letters denote statistically significant differences among exercised and non-exercised groups.

**Table 5:** Percentage of obesity, central obesity and elevated fat levels, according to gender and exercise.

	Non Exercised Elders		Exercised Elders	
	Men (n = 19)	Women (n = 27)	Men (n = 31)	Women (n = 25)
Overweight (%)	61.1%	55.6%	66.7%	56.5%
Obesity (%)	27.8%	25.9%	16.7%	13.0%
Central obesity (%)	52.6% * ^a^	81.5% * ^b^	36.7% * ^a^	40.0% * ^b^
High body fat level (%)	47.4%	37.0%	48.4%	44.0%

* *p* < 0.05 ^a,b^ Different letters denote statistically significant differences among exercised and non-exercised groups.
